# Structural determinants of the hyperalgesic activity of myotoxic Lys49-phospholipase A_2_

**DOI:** 10.1186/s40409-017-0099-6

**Published:** 2017-02-10

**Authors:** Vanessa Olzon Zambelli, Lucimara Chioato, Vanessa Pacciari Gutierrez, Richard John Ward, Yara Cury

**Affiliations:** 1 Butantan Institute, Special Laboratory for Pain and Signaling, Av. Vital Brazil, 1500, São Paulo, SP CEP 05503-900 Brazil; 2 Department of Chemistry, School of Philosophy, Sciences and Letters of Ribeirão Preto, University of São Paulo (USP), Ribeirão Preto, SP Brazil

**Keywords:** Lys49-Phospholipase A_2_, Hyperalgesia, Site-directed mutagenesis, Myotoxic effect, Edema, Membrane damage

## Abstract

**Background:**

Bothropstoxin-I (BthTx-I) is a Lys49-phospholipase A_2_ (Lys49-PLA_2_) from the venom of *Bothrops jararacussu,* which despite of the lack of catalytic activity induces myotoxicity, inflammation and pain. The C-terminal region of the Lys49-PLA_2_s is important for these effects; however, the amino acid residues that determine hyperalgesia and edema are unknown. The aim of this study was to characterize the structural determinants for the Lys49-PLA_2_-induced nociception and inflammation.

**Methods:**

Scanning alanine mutagenesis in the active-site and C-terminal regions of BthTx-I has been used to study the structural determinants of toxin activities. The R118A mutant was employed as this substitution decreases PLA_2_ myotoxicity. In addition, K115A and K116A mutants – which contribute to decrease cytotoxicity – and the K122A mutant – which decreases both myotoxicity and cytotoxicity – were also used. The H48Q mutant – which does not interfere with membrane damage or myotoxic activity – was used to evaluate if the PLA_2_ catalytic site is relevant for the non-catalytic PLA_2_-induced pain and inflammation. Wistar male rats received intraplantar injections with mutant PLA_2_. Subsequently, hyperalgesia and edema were evaluated by the paw pressure test and by a plethysmometer. Native and recombinant BthTx-I were used as controls.

**Results:**

Native and recombinant BthTx-I induced hyperalgesia and edema, which peaked at 2 h. The R118A mutant did not induce nociception or edema. The mutations K115A and K116A abolished hyperalgesia without interfering with edema. Finally, the K122A mutant did not induce hyperalgesia and presented a decreased inflammatory response.

**Conclusions:**

The results obtained with the BthTx-I mutants suggest, for the first time, that there are distinct residues responsible for the hyperalgesia and edema induced by BthTx-I. In addition, we also showed that cytolytic activity is essential for the hyperalgesic effect but not for edematogenic activity, corroborating previous data showing that edema and hyperalgesia can occur in a non-dependent manner. Understanding the structure-activity relationship in BthTx-I has opened new possibilities to discover the target for PLA_2_-induced pain.

## Background

Phospholipases A_2_ (PLA_2_; EC 3.1.1.4) are enzymes that hydrolyze the *sn*-2 acyl bond of glycerophospholipids, releasing free fatty acids and lysophospholipids [1]. Secretory PLA_2_s are found in a wide variety of biological fluids such as inflammatory exudates, and the venoms of arthropods, mollusks and snakes [2]. These enzymes are abundant in *Bothrops* snake venoms and display pharmacological activities characterized by myotoxic, neurotoxic, anticoagulant, hypotensive, hemolytic, platelet aggregation inhibition, bactericidal, pro-inflammatory and nociceptive effects [2–4]. A subfamily of class IIA PLA_2_s has been purified from the venoms of several viperid snakes, in which the Asp49 residue is replaced by Lys [5, 6]. These Ly49-PLA_2_s conserve the basic structural fold of this family of enzymes but lack catalytic activity.

While the Lys49-PLA_2_s do not show catalytic activity, in vitro studies showed they are able to disrupt liposome membranes and release their contents by a Ca^2+^-independent mechanism that does not involve hydrolysis of membrane phospholipids [7]. Despite the lack of catalytic activity, the in vivo activities of the Lys49-PLA_2_s include myonecrosis, bactericidal activity, local inflammation and pain [6, 8–13]. Chacur et al. [11] have demonstrated that the C-terminal cationic/hydrophobic sequence corresponding to amino acids 115–129 of a Lys49-PLA_2_ isolated from *Bothrops asper* is critical for the sensation of pain. This finding is supported by the demonstration that heparin partially neutralizes hyperalgesia induced by this toxin, and the direct induction of hyperalgesia by the peptide corresponding to amino acids 115–129, although having lower activity than the native toxin. Despite this evidence, the amino acids responsible for this effect are unknown.

Scanning alanine mutagenesis is a useful strategy to study the structural determinants of the activities of Lys49-PLA_2_. In this regard, Chioato et al. [14] have demonstrated that amino acid residues in C-terminal region of a Lys49-PLA_2_ from the venom of *Bothrops jararacussu* (BthTx-I) determine its biological activity. It has been demonstrated that the Lys^122^Ala mutant does not display myotoxic activity while Arg^115^Ala and Arg^116^Ala mutants do not display membrane-damaging activities. Moreover, His^48^Gln substitution, which eliminates any possible catalytic activity, does not influence the biological or membrane damaging proprieties of BthTx-I. Using these well-characterized functional point mutants in the active-site and C-terminal regions of the BthTx-I, we aimed to characterize the structural determinants for the Lys49-PLA_2_-induced nociception and inflammation, and more specifically, the edematogenic response.

## Methods

### Protein purification from crude venom

Bothropstoxin-I (BthTx-I) was purified from crude lyophilized *Bothrops jararacussu* venom using a single step cation-exchange chromatography as previously described [15]. The BthTx-I was eluted as a single peak and then dialyzed against 5 mM Tris–HCl, pH 7.5, for 36 h with buffer changes every 12 h and concentrated 10-fold by lyophilization. Protein purity was evaluated by silver staining of SDS-PAGE gels [16].

### Site directed mutagenesis

A full-length cDNA encoding BthTx-I has been previously isolated from *B. jararacussu* venom gland cDNA by RT-PCR (GenBank Acc. No. X78599) [17], and subcloned into the expression vector pET3-d [18]. The nucleotide sequencing has confirmed the construct in which Ser1 of the BthTx-I is preceded by a Met, and a stop codon immediately follows Cys133. After linearization of this construct with ScaI, site directed mutagenesis of the BthTx-I was performed by PCR mutagenesis [19] to introduce single mutations: Lys^115^ → Ala (K115A), Lys^116^ → Ala (K116A), Arg^118^ → Ala (R118A), Lys^122^ → Ala (K122A) and His^48^ → Gln (H48Q). The final PCR reactions were performed using oligonucleotides complementary to the vector sequences flanking the BthTx-I insert which contained restriction sites for XbaI (5′-extremity) and BamHI (3′-extremity). After digestion with these enzymes, the amplified fragments were subcloned into the equivalent sites in the expression vector pET3d and fully sequenced.

### Recombinant protein expression and purification

A 150-mL volume of growth medium (2.5 g yeast extract; 10 mM MgSO4; 15 μg/L chloramphenicol; 150 μg/L ampicillin; pH 7.5) was inoculated with *Escherichia coli* strain BL21(DE3)pLysS transformed with the native or mutant constructs in pET3d, and grown at 37 °C to an A600 of 0.6. Recombinant protein expression was induced by addition of 0.6 mM isopropylthiogalactoside, and the culture was grown for an additional period of 5 h. Inclusion bodies were isolated from bacterial pellets by repeated rounds of sonication in 20 mL of lysis buffer (50 mM Tris–HCl, pH 8.0; 1 mM EDTA; 0.4 M urea; 1% Triton X-100) followed by centrifugation at 12,000 *g*. The protocol for the solubilization and refolding of recombinant BthTx-I in the presence of a gel filtration medium was performed as previously described [18]. The refolded protein was applied directly to the cation exchange column and eluted as described earlier for the purification of the native BthTx-I from crude venom.

### Animals

Male Wistar rats, weighing between 170 and 190g were used. Rats were housed in a temperature-controlled (21 ± 2 °C) and light-controlled (12/12 h light/dark cycle) room with standard rodent rations and water *ad libitum*. All procedures were conducted in accordance with the guidelines of the International Association for the Study of Pain [20] and were approved by the Institutional Animal Care Committee of the Butantan Institute (CEUAIB, protocol number 118/2002).

### Pharmacological treatments

For the evaluation of hyperalgesia and allodynia, animals were injected with either 0.1 mL of sterile phosphate-buffered saline (PBS) solution (control animals) or 0.1 mL PBS containing the appropriate concentration of native, recombinant or mutant BthTx-I into the subplantar surface of one hind paw. For evaluation of edema, whereas toxins were injected into a hind paw and the PBS was administered in the contralateral paw.

### Evaluation of mechanical hyperalgesia (Randall and Selitto test)

An Ugo-Basile pressure apparatus [21] was used to assess pressure pain thresholds prior to, and again at different times after, intraplantar injection of native, recombinant, mutant BthTx-I or vehicle into the right hind paw. The contralateral paw was not injected. Testing was blind with respect to group designation. Briefly, a force (in *g*) with increasing magnitude was applied to the paw. The force needed to induce paw withdrawal was recorded as the pain threshold. To reduce stress, the rats were exposed to the testing procedure the day before the experiment, as previously described [22].

### Evaluation of low threshold mechanical allodynia (von Frey)

The von Frey test [23] was used to assess low-threshold mechanical pain thresholds prior to intraplantar injection of the toxins or PBS (control) at different periods of time later on. This test was performed as previously described in detail, using the modified up-down method [24]. Briefly, a logarithmic series of ten calibrated Semmes-Weinstein monofilaments (von Frey hairs, Stoelting, Wood Dale, USA) was applied to the right hind paw to determine the stimulus intensity threshold stiffness required to elicit a paw withdrawal response.

Log stiffness of the hairs is determined by log10 (milligrams × 10) and ranged from 3.61 (407 mg) to 5.18 (15.136 mg). Basal line assessment was initiated with the 2.041 mg hair. In the event of a paw withdrawal, the same hair was again presented 30–60 s later. If the response was again elicited, the 407 mg monofilament was presented. In the absence of a paw withdrawal response to the 407 mg stimulus, the next stronger monofilament was presented (692 mg). The monofilament that elicited a clear response was recorded, and was presented once again 30–60 s later. If the animal withdrew its paw on two consecutive trials with the same stiffness value, no further von Frey hairs were tested.

However, in the absence of a response to the initial 2.041 mg monofilament, presentation of monofilaments continued in ascending order until two consecutive responses were elicited from the same monofilament. All single responses were recorded, but assessment was complete only after two consecutive responses were elicited from the same monofilament. In instances when rats failed to respond, the strongest stimulus (15.136 mg) was considered to be the cut-off value.

Responses that occurred to the weakest stimulus (407 mg) were assigned the lower cut-off value for that time point. To reduce stress, rats were habituated to the experimental environment on each of the four days before experiments. Behavioral responses were used to calculate the 50% paw withdrawal threshold (absolute threshold) by fitting a Gaussian integral psychometric function using a maximum-likelihood fitting method. This fitting method allows parametric analyses [24, 25].

### Evaluation of edema

The volume increase (edema) of paws up to the tibiotarsal articulation was plethysmographically measured before toxin or PBS (control) injection and subsequently at chosen time intervals according to the method of Van Arman et al. [26]. The percentage of increase in paw volume was determined for each paw. The difference between the values obtained for both paws was used as a measure of edema.

### Statistical analysis

The results are presented as the mean ± SEM. The statistical evaluation of data was conducted using a two-way analysis of variance (ANOVA) with *post-hoc* testing by Tukey. A value of *p* < 0.05 was considered significant.

## Results

### Characterization of hyperalgesia and edema induced by native and recombinant bothropstoxin-I

**Fig. 1:**
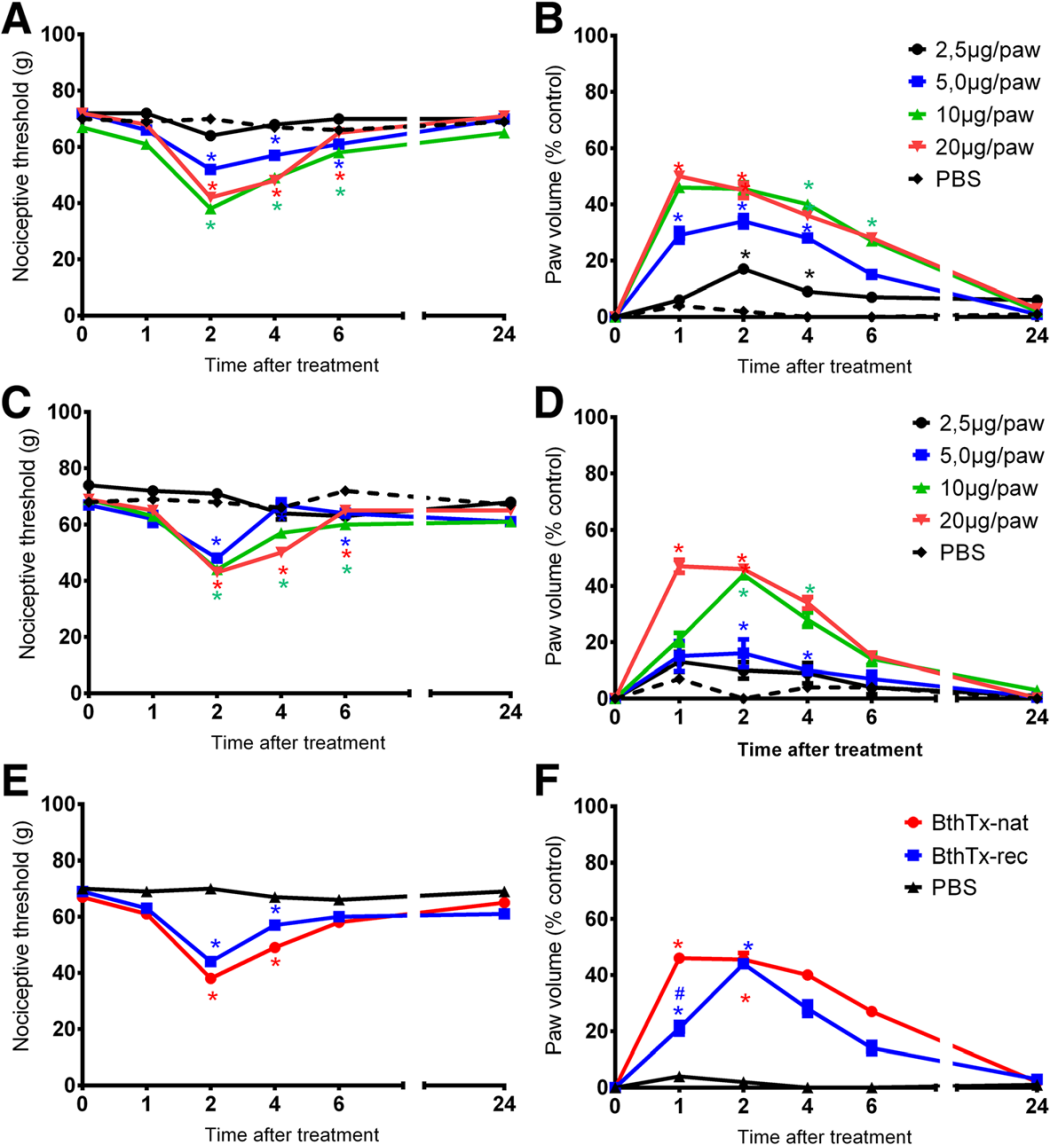
Effect of native and recombinant bothropstoxin I injection on the pain threshold and paw volume. **a** Effect of the native-BthTx intraplantar injection on the pain threshold and **b** on paw volume, at different doses. **c** Effect of recombinant BthTx-I intraplantar injection on the pain threshold and **d** on paw volume, at different doses. **e** Comparison between native and recombinant BthTx-I hyperalgesic and **f** edematogenic effects. The pain threshold of the animals was determined in rat hind paw before at different times after the intraplantar injection of PBS (control group) or toxins. Sensitivity to pain was measured as the threshold response to pressure and expressed as *g*. The edema was determined by an increase in the volume in the right hind paw of rats compared with the control contralateral paw. The paw volume was obtained pletsmografically. Each point represents the mean ± SEM of six animals. *Significantly different from mean values before venom injection and #different from BthTx-I-recombinant (BthTx-I rec) (*p <* 0.05)

Intraplantar injection of 2.5 μg of native BthTx-I did not alter the sensitivity to pressure pain, as measured by the Randall and Sellito test. In contrast, doses of 5, 10 and 20 μg/paw decreased the pain threshold (28%, 43% and 42%, respectively) of the animals as compared to the basal values. The peak of mechanical hyperalgesia was detected at 2 h. Intraplantar PBS injection (vehicle control) did not modify the pain threshold of the animals (Fig. 1a). Native BthTx-I also induced a significant edematogenic response when injected at 10 and 20 μg/paw. The dose of 10 μg/paw caused maximal response at 2 h (44%), while a dose of 20 μg/paw showed a peak effect 1 h (47%) after toxin administration, decreasing thereafter and completely disappearing within 24 h (Fig. 1b).

In order to evaluate whether the recombinant form of BthTx-I induces hyperalgesia and edema, a dose response curve was performed for recombinant BthTx-I. As is the case for the native toxin, the intraplantar injection of 2.5 μg/paw of recombinant BthTx-I did not induce significant alteration in the pain threshold of the animals. Doses of 5, 10 and 20 μg/paw significantly decreased the pain threshold as compared with baseline, 2h after toxin injection (27%, 42% and 41%, to 5, 10 and 20 μg/paw, respectively). Injection of PBS (control) did not modify the pain threshold of the animals (Fig. 1c). Moreover, the doses of 5, 10 and 20 μg/paw caused a significant edematogenic response. The peak of the edematogenic response was detected 2 h after injection of 5 μg/paw (34%) of BthTx-I, or 1 h after the administration of 10 (46%) or 20 μg/paw (50%) of the toxin. Intraplantar injection of PBS (vehicle control) did not alter the paw volume of the animals (Fig. 1d).

For comparative analysis, native and recombinant BthTx-I at 10 μg/paw induced similar intensity of hyperalgesia (Fig. 1e). Although the recombinant BthTx-I did not induce edema at the same magnitude as native at 1 h, both toxins induced similar edema 2 h after the treatment (Fig. 1f). Therefore, the dose of 10 μg/paw was selected for subsequent studies.

### Effect of BthTx-I site-directed mutagenesis on rat pain threshold and paw volume

**Fig. 2:**
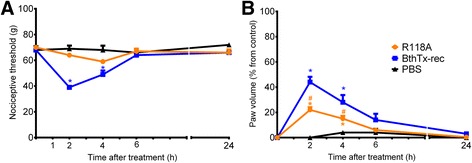
Effect of BthTx-I R118A (Arg118 → Ala) mutagenesis on rat pain threshold and paw volume. Decrease in **a** threshold response and **b** paw volume were determined in rat hind paw before and 2, 4, 6 and 24 h after the intraplantar injection of PBS (control group) or R118A, or recombinant BthTx-I. The paw volume was obtained pletsmografically. Sensitivity to pain was measured as the threshold response to pressure and expressed as *g*. The paw volume was obtained pletsmografically. Each point represents the mean ± SEM of six animals. *Significantly different from mean values before venom injection and #different from BthTx-I-recombinant (BthTx-I rec) (*p <* 0.05)

In order to investigate whether the residues involved in the determination of myotoxic activities were also critical for hyperalgesia and edema, the BthTx-I mutant R118A was tested. BthTx-I-induced hyperalgesia was blocked by R118A mutation (in which the myotoxic activity is reduced) (Fig. 2a). In addition, the R118A mutation significantly decreased the edema induced by recombinant BthTx-I (Fig. 2b).

**Fig. 3:**
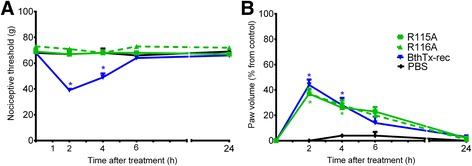
Effect of BthTx-I K115A and K116A (Lys115 → Ala and Lys116 → Ala) mutagenesis on rat pain threshold and paw volume. Decrease in **a** threshold response and **b** paw volume were determined in rat hind paw before and 2, 4, 6 and 24 h after the intraplantar injection of PBS (control group) or R115A, or R116A, or recombinant BthTx-I. Sensitivity to pain was measured as the threshold response to pressure and expressed as *g*. The paw volume was obtained pletsmografically. Each point represents the mean ± SEM of six animals. *Significantly different from mean values before venom injection (*p <* 0.05)

In order to investigate whether the residues involved in the BthTx-I membrane damaging activities were also critical for hyperalgesia and edema, the K115A and K116A mutants were tested. BthTx-I induced hyperalgesia was completely abolished by both K115A and K116A (which reduce the membrane-damaging activity) (Fig 3a).

**Fig. 4:**
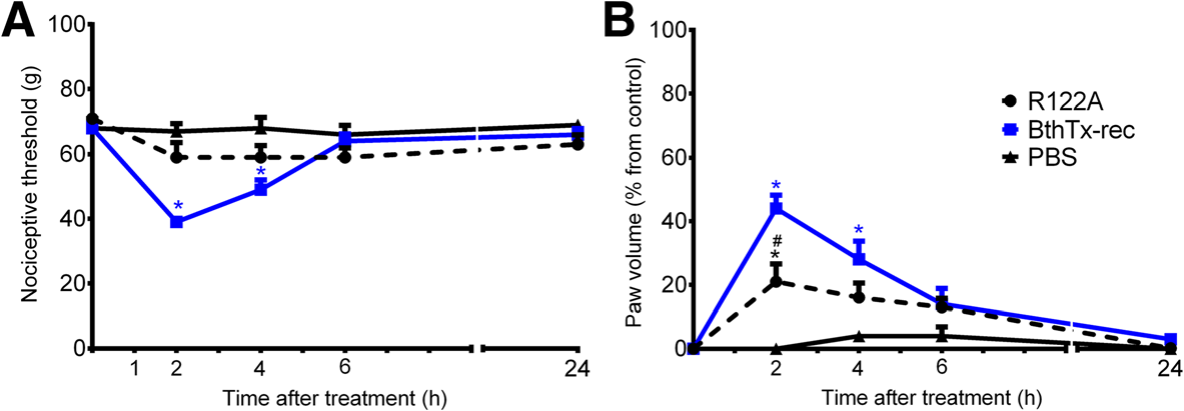
Effect of BthTx-I K122A (Lys122 → Ala) mutagenesis on rat pain threshold and paw volume. Decrease in **a** threshold response and **b** paw volume were determined in rat hind paw before and 2, 4, 6 and 24 h after the intraplantar injection of PBS (control group) or R122A, or recombinant BthTx-I. Sensitivity to pain was measured as the threshold response to pressure and expressed as *g*. The paw volume was obtained pletsmografically. Each point represents the mean ± SEM of six animals. *Significantly different from mean values before venom injection (*p <* 0.05)

The K122A mutation, which significantly reduces both myotoxic and membrane damaging activities also reduced the rat hind paw hyperalgesia induced by BthTx-I (Fig. 4a). The K122A mutation also significantly decreased the edema induced by recombinant BthTx-I (Fig. 4b).

**Fig. 5:**
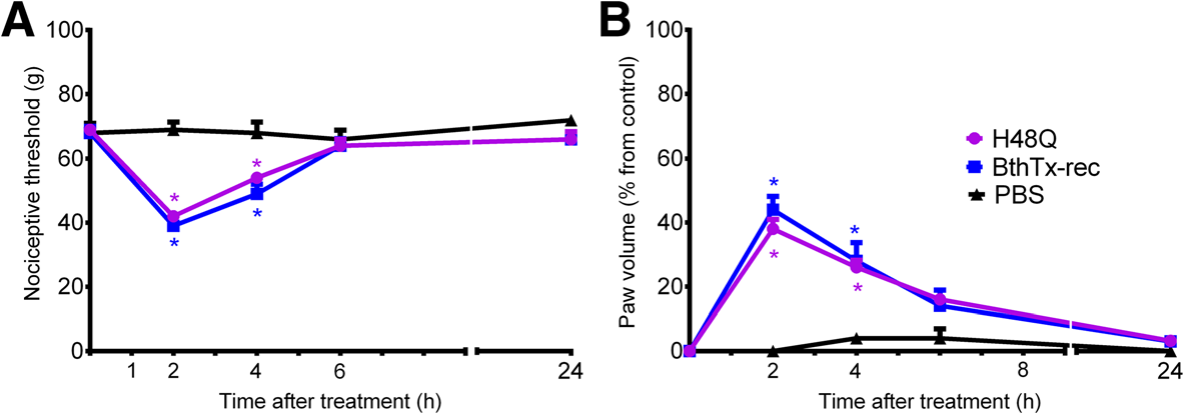
Effect of H48Q (BthTx-I His48 → Gln) mutagenesis on rat pain threshold and paw volume. Decrease in **a** threshold response and **b** paw volume were determined in rat hind paw before and 2, 4, 6 and 24 h after the intraplantar injection of PBS (control group) or H48Q, or recombinant BthTx-I. Sensitivity to pain was measured as the threshold response to pressure and expressed as *g*. The paw volume was obtained pletsmografically. Each point represents the mean ± SEM of six animals. *Significantly different from mean values before venom injection (*p <* 0.05)

The H48Q mutation eliminates catalytic activity in class II PLA_2_s, and even though no catalytic activity is detected in BthTx-I, this mutant was used as a control to eliminate the possibility that the observed effects are the result of phospholipid hydrolysis. The H48Q mutation did not modify the hyperalgesia or edematogenic response induced by BthTx-I (Fig. 5a and Fig 5b).

### Characterization of allodynia induced by native and recombinant bothropstoxin-I

**Fig 6:**
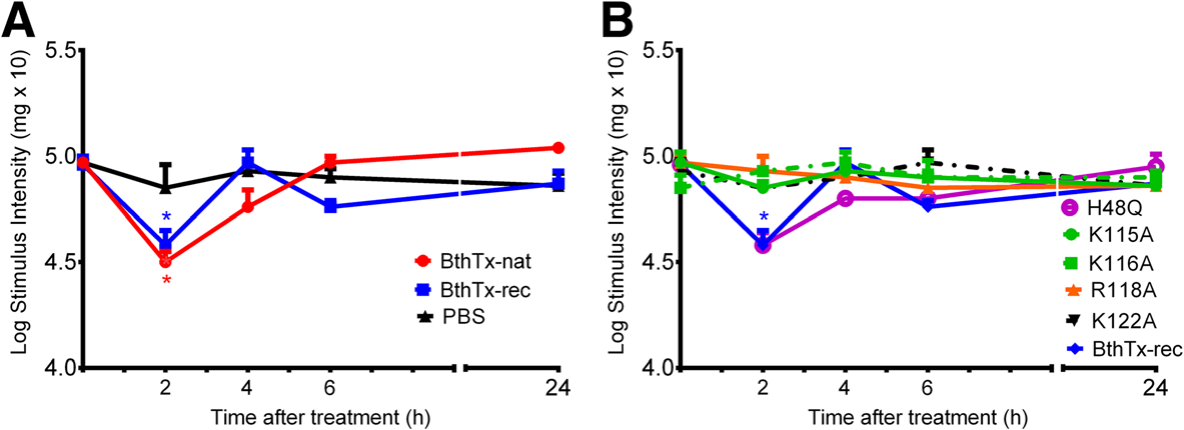
Characterization of allodynia induced by native and recombinant BthTx-I (**a**) and mutants BthTx-I (**b**). Decrease in tactile threshold was evaluated by von Frey test, before and 2, 4, 6 and 24 h after the intraplantar injection of PBS (control group), native, recombinant BthTx-I (**a**) or BthTx-I mutants (**b**). Sensitivity to pain was measured as the threshold response to tactile and expressed as *g* Log (mg × 10). Each point represents the mean ± SEM of six animals. *Significantly different from mean values before venom injection (*p <* 0.05)

Intraplantar injection (10 μg/paw) of either the native or recombinant lowered withdrawal thresholds, as measured by the von Frey test. This effect was observed 2 h after native Bthtx-I (65%) or recombinant BthTx-I (58%) injection, and completely disappeared within 24 h. The injection of PBS (control group) did not modify the pain threshold of the animals (Fig. 6a).

#### Effect of BthTx-I site-directed mutagenesis on allodynia

The intraplantar administration of H48Q (10 μg/paw) lowered withdrawal thresholds, comparing with the baseline. The allodynic effect was observed 2 h after toxin injection (58%). The other BthTx-I mutants (Arg118 → Ala, Arg115 → Ala, Arg116 → Ala, Arg122 → Ala) did not alter the threshold (Fig. 6b).

## Discussion

Site-directed mutagenesis studies can identify the structural determinants for biological activities of venom PLA_2_s. In this study, we demonstrate for the first time the involvement of amino acids in the C-terminal region in the nociceptive activity of the BthTx-I, a non-catalytic Lys49-PLA_2_ from *Bothrops jararacussu* venom. In addition, we have demonstrated that the same residues that are determinants of myotoxicity of the BthTx-I are also involved in hyperalgesia and inflammation, whereas the residues responsible for the cytolytic activity only contribute to the nociceptive effect of the protein.

In the experimental procedures, the recombinant BthTx-I was used as control for all the behavior experiments performed. Circular dichroism spectroscopic analysis has previously confirmed that the protein secondary structures were preserved in the recombinant molecule, as well as its biological activities (myotoxicity and cytolytic effects). Here we have shown that the recombinant BthTx-I displays hyperalgesic and edematogenic responses with similar onset, intensity and time course to those observed for the native BthTx-I. The edematogenic activity of the native BthTx-1 has been previously demonstrated [27, 28]. However, to the best of our knowledge, this is the first report showing that BthTx-I induces hyperalgesia in an experimental model of pain evaluation.

It is well established that the PLA_2_ component contributes to the local effects induced by the *Bothrops* snake envenomation. Our group has previously demonstrated that both the Lys49-PLA_2_ that is devoid of catalytic activity, and the catalytically active Asp49-PLA_2_ from *Bothrops asper* venom cause significant local hyperalgesia in rat paws after intraplantar injection. The hyperalgesic effect induced by these PLA_2_s is mediated by biogenic amines, bradykinin, cytokines, prostaglandins and sympathomimetic amines that may interact and be sequentially released [11].

Because Lys49-PLA_2_s are unable to catalyze phospholipid hydrolysis, their toxicity has been explained by some mechanisms that differ from that of their catalytically active PLA_2_ counterparts. It has been suggested that the C-terminal region of Lys49-PLA_2_s from *Bothrops* venoms is critical for their biological activities [14, 29–33]. The nociceptive effect of Lys49-PLA_2_ has also been investigated, and we have previously shown that the C-terminal region of the Lys49-PLA_2_ from *Bothrops asper* venom is important for hyperalgesia, since the intraplantar injection of a peptide corresponding to amino acids 115–119 in the C-terminal region of the protein induces hyperalgesia in rats. In contrast, a C-terminal peptide derived from the same region of the Asp49-PLA_2_ did not show any nociceptive effect [11].

It has been demonstrated that the C-terminal region of the Lys-PLA_2_ region is also responsible for cytolytic, edematogenic, and myotoxic activities of this PLA_2_ [31, 34, 35]. Furthermore, this C-terminal region is also endowed with bactericidal activity, and a peptide corresponding to residues 115–119 of the BthTx-I reproduces the antimicrobial effect of the role Lys49-PLA_2_ [36, 37]. Taken together these data indicate that the C-terminal region may have an important role in the biological effects of venom-derived Lys49-PLA_2_. However, crystallographic and site-directed mutagenesis studies have suggested that additional residues, other than those located at C-terminal, participate in Lys49-PLA_2_ toxicity, and Lys20 is also critical for the myotoxic activity of this molecule [29, 32].

Despite the importance of the C-terminal region for the different biological activities of the Lys49-PLA_2_s, the membrane damaging, myotoxic and bactericidal activities have distinct structural determinants. This suggestion is based on scanning alanine mutagenesis studies showing that the structural determinants of the bactericidal activity are more extensive and only partially overlap with the structural determinants of the myotoxic and cytolytic activities [14, 29]. However, the results obtained in the present study indicate a degree of similarity in the structural determinants involved in the myotoxic, cytolytic, hyperalgesic and edematogenic effects. This observation is based on data showing that:

- The same residue responsible for the myotoxic activity (R118) also contributes to the edematogenic and hyperalgesic responses induced by BthTx-I [14].
- Residues 115 and 116, which are determinants for the Ca^2+^-independent membrane damaging activity of the BthTx-I, are also critical for the hyperalgesic effect of this Lys-PLA_2_, but not for the edematogenic response [12, 14, 29].
- The R122A, which contributes to both the myotoxic and cytolytic activities of BthTX-I, is also important for hyperalgesia and edema.

The results obtained in this study indicate that the BthTx-I-induced hyperalgesia depends on the main biological activities of this Lys-PLA_2_, since elimination of the myotoxic and cytolytic activities also abolished hyperalgesia. In contrast, the edematogenic response is less dependent on the cytolytic effects of BthTx-I, since the elimination of myotoxicity interfered with this activity. These data also suggest that hyperalgesia and edema caused by this Lys-PLA_2_s are not directly correlated.

Previous data from our group have demonstrated that different mediators are involved in the genesis of hyperalgesia and edema caused by Lys49 and Asp49-PLA_2_s from *Bothrops* snake venoms, reinforcing the suggestion that hyperalgesia and edema induced by BthTx-I are not directly dependent [11].

The mechanisms that contribute to myotoxicity and to hyperalgesia and edema of the Lys49-PLA_2_s are not yet characterized. Preliminary data have demonstrated that the R118 mutant, which is devoid of myotoxic activity, induces decreased edematogenic activity, and a substantial drop in the number of local neutrophils (Zambelli and Cury, 2004, personal communication), confirming the importance of myotoxicity to the inflammatory response caused by this molecule. Despite the evidences indicating a possible correlation between myotoxicity and inflammation, we should also consider that a reduction in myotoxicity abrogates hyperalgesia by a mechanism independent of inflammation. The Lys49-PLA_2_ from *B. asper* induces a ATP and K^+^ release from C2C12 myotubes in culture and from mouse muscles [38]. It has been demonstrated that these mediators can directly induce pain by activating purinergic receptors or inducing membrane depolarization of peripheral sensory nerves [38–40]. Therefore, a direct link between myotoxicity and pain generation may exist.

The data showing that the residues 115 and 116 are critical for hyperalgesia, but not for edema formation, also adds evidence that nociception and edema are not directly correlated, and may have distinct structural determinants. Although there is no data available to explain how these residues contribute exclusively for hyperalgesia, these mutations are involved in the BthTx-I cytolytic activity and a direct effect of cytotoxicity on pain generation is a possibility. Further experiments are necessary to investigate this hypothesis.

To further characterize the structural determinants involved in BthTx-I-induced hyperalgesia, and to evaluate whether a putative residual catalytic activity of the Lys49-PLA_2_ could play a role in the hyperalgesia and the edema induced by BthTx-I, we tested the H48Q mutant in our experimental conditions. The hydrolytic mechanism of Asp49-PLA_2_s involves the His48 in the catalytic site that activates a conserved water molecule, thereby initiating the nucleophilic attack on the *sn*-2 position of the phospholipid substrate and the H48Q mutation abolishes this activity [41, 42]. Here we observe that H48Q mutation does not interfere with the hyperalgesic and edematogenic effects induced by the BthTx-I, confirming that only the C-terminal amino acids are relevant for the BthTx-I pharmacological activities. It is important to highlight that the H48Q mutation does not alter the myotoxicity or the membrane damaging effects of the BthTx-I [14].

The data presented here suggest that residues present in the C-terminal region of the BthTx-I are important for hyperalgesia and edema. Previous studies have demonstrated that the synthetic peptide 115–129 was unable to mimic some effects of the whole toxin, such as in vitro and in vivo myotoxicity, and toxicity against epithelial cells and erythrocytes [30, 37]. These findings indicate that other residues present in the whole molecule or the quaternary structure of Lys49-PLA_2_s are critical for the for the biological activity of these molecules [32, 43]. However, the absence of toxicity observed for the synthetic peptide 115–129 do not exclude the participation of C-terminal residues in the pharmacological effects evaluated.

## Conclusions

In conclusion, the results obtained with the BthTx-I mutants suggest, for the first time, that there are distinct residues responsible for the hyperalgesia and edema induced by BthTx-I. In addition, we also showed that the cytolytic activity is essential for the hyperalgesic effect but not for edematogenic activity, reinforcing previous data showing that edema and hyperalgesia can occur independently. A better understanding about the structure-activity relationship can open new avenues of investigation to identify the target for PLA_2_-induced pain.

## Abbreviations

ANOVA: analysis of variance
BthTx-I: bothropstoxin-I
cDNA: complementary deoxyribonucleic acid
EDTA: ethylenediaminetetraacetic acid
H48Q: His48 → Gln
K115A: Lys115 → Ala
K116A: Lys116 → Ala
K122A: Lys122 → Ala
MgSO_4_: magnesium sulfate
PLA_2_: phospholipase A_2_
PLA_2_-Lys49: phospholipase A_2_ with a Lys at position 49
R118A: Arg118 → Ala
RT-PCR: reverse transcriptase polymerase chain reaction
Tris–HCl: Tris hydrochloride
